# Agreement Between Predicted and Actual Measured Ablation Depth After FS-LASIK Using Different Rotating Scheimpflug Cameras and OCT

**DOI:** 10.3389/fmed.2022.907334

**Published:** 2022-05-19

**Authors:** Hao Chen, Ziqian Wang, Kunke Li, Yiran Wang, Xin Li, Lan Du, Meimin Lin, Giacomo Savini, Qinmei Wang, Ayong Yu, Sisi Chen

**Affiliations:** ^1^School of Ophthalmology and Optometry and Eye Hospital, Wenzhou Medical University, Wenzhou, China; ^2^Shenzhen Eye Hospital, Shenzhen Key Ophthalmic Laboratory, Jinan University, Shenzhen, China; ^3^IRCCS G.B. Bietti Foundation, Rome, Italy

**Keywords:** ablation depth, laser *in situ* keratomileusis, femtosecond laser, difference, agreement

## Abstract

**Purpose:**

To compare the predicted ablation depth (AD) with the postoperatively measured corneal ablation depth (postop-AD) at central, paracentral, and midperipheral locations using two rotating Scheimpflug analyzers and a Fourier-domain optical coherence tomographer in eyes that underwent femtosecond laser-assisted LASIK (FS-LASIK).

**Methods:**

The values of corneal thickness were measured preoperatively and postoperatively at one and three months. The difference between preoperative and postoperative was defined as postop-AD. Measurements were performed at the corneal vertex and mid-peripheral area. The mid-peripheral corneal thickness was measured at the superior, inferior, nasal, and temporal locations at a distance of 1.0 or 2.5 mm from the corneal vertex. The predicted AD was calculated by ORK-CAM software (Schwind eye tech-solutions GmbH, Kleinostheim, Germany), and the difference between the predicted AD and postop-AD was defined as Δ-AD. Paired *t-*test analysis was employed to evaluate the differences, agreement was assessed by the Bland-Altman method.

**Results:**

Forty-two eyes of 42 patients were investigated. At one month, the predicted AD in the central and paracentral areas was underestimated by the Pentacam HR (Oculus, Wetzlar, Germany), Sirius (Costruzione Strumenti Oftalmici, Florence, Italy) and RTVue OCT (Optovue Inc., Freemont, CA, United States), whereas Δ-AD was negative as established by all devices and predominantly statistically significant. The Δ-AD values approximated zero at three months. The mean difference of Δ-AD at three months at the corneal vertex was 0.67 ± 9.39 mm, −7.92 ± 9.05 mm and −1.36 ± 8.31 mm, respectively. The mid-peripheral measurements had positive values at one month and even more highly positive at three months (with statistically significant differences in most of the cases). The agreement between the predicted and postop-AD was moderate with all devices, but slightly better with RTVue.

**Conclusion:**

The predicted AD seems to be underestimated in the central and paracentral corneal area and overestimated in the mid-periphery.

**Translational Relevance:**

The study could help to partly explain and prevent the refractive errors after FS-LASIK.

## Introduction

Femtosecond laser-assisted laser *in situ* keratomileusis (FS-LASIK) offers several advantages over microkeratome-assisted LASIK and is now considered one of the safest and most effective corneal refractive surgical procedures. However, to increase safety and reduce the risk of iatrogenic corneal ectasia, the residual stromal thickness should be higher than 300 μm (1, 2). Furthermore, to promote surgical effectiveness, an accurately determined quantity of corneal stroma should be ablated to avoid over- or undercorrection.

Several studies, which assessed the agreement between the predicted ablation depth (AD) of the laser treatment and the postoperatively measured ablation depth (postop-AD), obtained contradictory results. The postop-AD in some investigations was higher than the predicted AD, whereas opposite results and a lack of statistically significant differences were also reported (3–11). There are several confounding variables that may justify this observation, such as different platforms, different modalities of ablation pattern, even different surgeries.

Several non-contact optical devices can be used for performing the aforementioned surgical procedures, which produced accurate and precise measurements of corneal thickness (12, 13). The possibility of simultaneously measuring the central and mid-peripheral corneal thickness and the high repeatability and reproducibility made them suitable for examinations on the amount of tissue ablation after excimer laser surgery.

In this study, we used a rotating Scheimpflug camera (Pentacam HR, Oculus, Wetzlar, Germany), a rotating Scheimpflug camera combined with a Placido disk corneal topographer (Sirius, Costruzione Strumenti Oftalmici, Florence, Italy), and a Fourier-domain optical coherence tomographer (RTVue OCT, Optovue Inc., Fremont, CA, United States) to measure the corneal thickness and evaluate the agreement between the predicted AD and the postop-AD.

## Patients and Methods

### Subjects

In this prospective study, we enrolled consecutive subjects undergoing FS-LASIK for myopia and myopic astigmatism correction. The following inclusion criteria were applied: age over 18, no contraindications to FS-LASIK, corrected distance visual acuity (CDVA) lower than 0.1 LogMAR, no history of wearing soft contact lenses in the last two weeks or rigid contact lenses in the last four weeks, no history of ocular trauma, surgery, or long-term eye medications. The exclusion criteria were as follows: any ophthalmic disease (keratoconus, glaucoma, retinal detachment, *etc*.), any intraoperative or postoperative complication and refractive regression ≥ −0.5 diopters (D) occurring during the follow-up period. Each patient was informed of the purpose of the study and signed a written consent form. The study protocol was approved by the Ethics Committee of the Eye Hospital of Wenzhou Medical University (Wenzhou, Zhejiang, China). The study methods adhered to the tenets of the Declaration of Helsinki.

### Instruments

In this study, we used three devices to measure patients’ corneal thickness: Pentacam HR, Sirius, and RTVue OCT. The Pentacam HR utilizes a rotating Scheimpflug camera to provide a 3-dimensional scan of the anterior segment of the eye with good repeatability and reproducibility (14). The slit-light source (diode-emitting blue light at 475 nm) rotates around the optical axis of the eye. It can capture 25 slit images of the anterior segment and obtain 138,000 true elevation points.

Sirius is based on the combination of a rotating Scheimpflug camera and a Placido disk topographer with 22 rings. A full scanning acquires a series of 25 Scheimpflug images and one Placido top-view image. It allows a comprehensive analysis of the topography and elevation of the anterior and posterior corneal surface and full corneal thickness (15).

The RTVue OCT is a Fourier-domain optical coherence tomography (FD-OCT) with 5 μm of depth resolution in the tissue. Its measurements had good repeatability and reproducibility in several previous studies (12, 16, 17). A super-luminescence diode, which emits light with a 50-nm bandwidth, centered at 830 nm, is used as a low-coherence light source. A corneal-anterior module lens with low magnification is added to the RTVue when imaging the anterior segment, which enables corneal thickness measurement.

### Surgical Technique

Each patient underwent ordinary bilateral FS-LASIK by the same surgeon using Intralase (iFS, Advanced Medical Optics, Santa Ana, CA, United States) 150-K femtosecond laser and the Amaris 750S excimer laser (Schwind eye tech-solutions GmbH, Kleinostheim, Germany). Flap thickness was set at 95, 100, or 105 μm. The predicted AD of each eye was recorded. After the surgery, the patients were prescribed 0.5% levofloxacin to be taken four times a day for the first three days, 0.1% fluorometholone to be taken four times a day for the first week and then tapered off during the following one month, and preservative-free artificial tears to be taken every hour for the first day and then tapered off during the following three months.

### Measurements

Corneal thickness measurements were performed preoperatively and one and three months postoperatively by an experienced examiner. They were conducted by a single well-trained operator experienced in using all the three devices. All eyes were measured without dilation in a dim room between 10 AM and 5 PM to minimize the effects of diurnal variation in corneal shape and thickness (18). For each subject, the order of the three instruments was arranged randomly and the interval between different instruments was just few minutes. Before measurements, all subjects were positioned in the headrest and asked to fixate on the target; then they were instructed to completely blink once before scanning to spread an optically smooth tear film over the cornea. Only scans with an “examination quality specification” of “OK” were retained for analysis, whereas the ones with insufficient quality were deleted and repeated. For each device, two consecutive measurements were performed, which were then averaged to determine the mean value of the corneal thickness obtained by each device.

The corneal thickness measurements obtained by each instrument included central corneal thickness (CCT) and mid-peripheral corneal thickness (MPCT). The CCT was measured at the corneal vertex and the MPCT was measured at the superior, inferior, nasal, and temporal locations at a distance of 1.0 or 2.5 mm from the corneal vertex (*i.e*., along a ring with a diameter of, respectively, 2.0 and 5.0 mm). Namely, in addition to CCT, eight categories of MPCT (including four directions and two distances) were chosen for analysis and were defined as: CT_S–1mm_, CT_I–1mm_, CT_N–1mm_, CT_T–1mm_, CT_S–2_._5mm_, CT_I–2_._5mm_, CT_N–2_._5mm_, and CT_T–2_._5mm_.

Schwind ORK-CAM software was used to obtain a full corneal graph of the predicted AD by entering the planned dioptric correction, corneal power, and optical zone diameter. Then we used the mouse to manually record the predicted AD at the optical axis, and at the superior, inferior, nasal, and temporal locations at a distance of 1.0 or 2.5 mm from the optical axis. They were defined as predicted AD_C_, AD_S–1mm_, AD_I–1mm_, AD_N–1mm_, AD_T–1mm,_ AD_S–2_._5mm_, AD_I–2_._5mm_, AD_N–2_._5mm_, and AD_T–2_._5mm_. The difference between the preoperative and postoperative corneal thickness were denoted as postop-AD, including postop-AD_C_, postop-AD_S–1mm_, postop-AD_I–1mm_, postop-AD_N–1mm_, postop-AD_T–1mm_, postop-AD_S–2_._5mm_, postop-AD_I–2_._5mm_, postop-AD_N–2_._5mm_, and postop-AD_T–2_._5mm_. The difference between the predicted AD and postop-AD (ΔAD) was calculated as predicted AD minus postop-AD.

### Statistical Analysis

Since ocular measurements are more alike between fellow eyes and cannot thus be treated as independent, in this study the parameter values of only the right eye of each patient were subjected to statistical analysis (19). All statistical analyses were performed using SPSS21.0 (SPSS, Inc., Chicago, IL, United States) and MedCalc (Version 11.4.2.0, MedCalc Software, Inc., Mariakerke, Belgium). A *P*-value less than 0.05 was considered statistically significant. The Kolmogorov-Smirnov test was employed to evaluate the normal distribution of data. The difference between the predicted AD and postop-AD was analyzed by a paired *t-*test. The agreement was evaluated using Bland-Altman plots, in which the differences between the predicted AD and postop-AD were plotted against their means (20). The 95% limits of agreements (LoA) were defined as the mean difference ± 2 standard deviations of the differences.

## Results

Fifty-four eyes of 54 patients were initially enrolled, but due to missing follow-up of 12 patients, only 42 eyes were analyzed at both one and three months postoperatively. Hence this sample was investigated throughout the whole study. The mean age of the 42 subjects was 25.1 ± 5.0 years (range from 18 to 39), and the mean spherical equivalent correction was −5.84 ± 1.89 D (range from −3.13 to −11.88 D). The mean spherical correction was −5.45 ± 1.85 D (range from −2.25 to −11.25 D) and mean cylindrical correction was −0.79 ± 0.60 D (range from 0.00 to −2.50 D). The mean optical zone and treat zone was 6.7 ± 0.4 mm (range from 5.6 to 7.4 mm) and 7.7 ± 0.39 mm (range from 6.76 to 8.48 mm), respectively. The mean predicted AD was 93.5 ± 18 μm (range from 51 to 121 μm). No intraoperative and postoperative complications were detected.

The data of the comparison and agreement between the predicted AD and postop-AD at one month are listed in Supplementary Tables 1–3. Supplementary Tables 4–6 present the data obtained at three months postoperatively. The procedures with all studied devices resulted in negative central ΔAD at one month, with an average value of around 10 microns, indicating an underestimation of the predicted AD with respect to the postop-AD. The difference was more evident in the measurements conducted by Sirius (Supplementary Table 2), which was statistically significant (*P* < 0.0001) for all devices. At three months, the central ΔAD became closer to zero with Pentacam and RTVue OCT, where it lost its statistical significance, and decreased with Sirius, because of progressive thickening of postoperative CCT.

**FIGURE 1:**
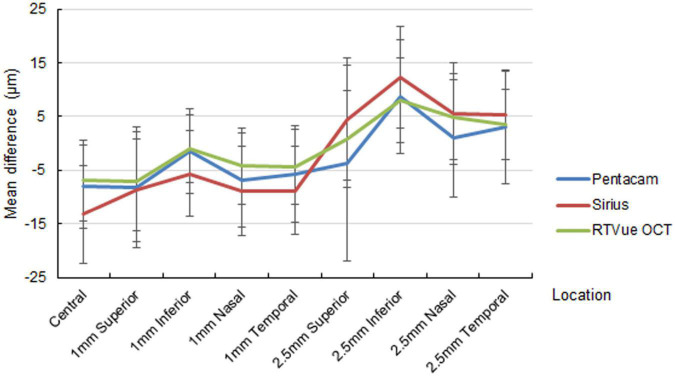
Mean difference of the predicted ablation depth against actual measured corneal ablation depth determined by Pentacam HR (blue line), Sirius (red line), and RTVue OCT (green line) at one month postoperatively.

**FIGURE 2:**
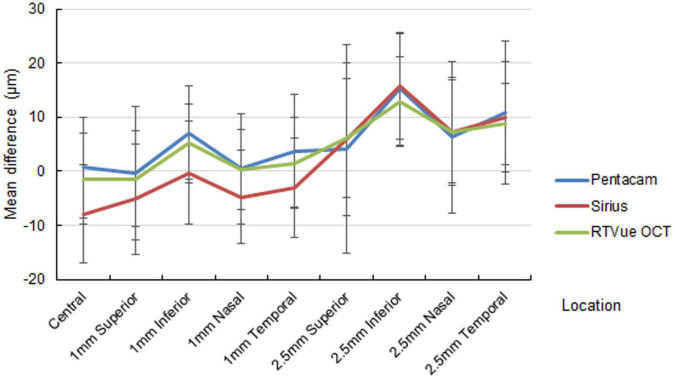
Mean difference of the predicted ablation depth against actual measured corneal ablation depth determined by Pentacam HR (blue line), Sirius (red line), and RTVue OCT (green line) at three months postoperatively.

The out-of-center measurements of the one-month paracentral values (2-mm diameter) were negative for all devices, with ΔAD ranging between −1.01 ± 6.26 and −8.82 ± 8.31 μm (Figure 1). Conversely, the mid-peripheral values (5-mm diameter were slightly positive, with the only exception of the superior location by Pentacam HR. At three months, the mean differences at the paracentral locations turned from negative to positive for Pentacam HR and RTVue, and approximated zero for Sirius; the mean differences at the mid-peripheral locations were more highly positive than at one month, often exceeding 10 μm (Figure 2).

Paracentral ΔAD was statistically significant in most locations for all devices at one month, but this significance was absent at three months in some locations, especially as determined by RTVue. On the other hand, midperipheral ΔAD was statistically significant in most locations for all devices at one month, with higher significance at three months.

The data obtained by the Bland-Altman method (Supplementary Tables 1–6 and Supplementary Figure 1–6) show only moderate agreement between the predicted AD and the postop-AD, indicating that the difference was not only statistically but also clinically significant.

## Discussion

Corneal ectasia is one of the most severe complications of corneal refractive surgery. To minimize its occurrence, the preservation of at least 300 μm of residual stromal thickness is commonly considered necessary (1, 2). The percent tissue altered (PTA) is another important risk factor for corneal ectasia (21, 22). An accurate estimation of the residual stromal thickness depends on an precise assessment of AD.

Several earlier studies have been conducted to evaluate the accuracy of the predicted AD. Some authors measured the preoperative and the postoperative corneal thickness and defined this difference as the postop-AD, whereas others intraoperatively measured the stromal bed thickness after lifting the flap and after the ablation. The former ones used different technologies, such as OCT, Scheimpflug tomography, ultrasound pachymetry, specular microscopy, and confocal microscopy (6, 10, 11, 23, 24). The latter conducted ultrasound pachymetry and optical coherence pachymetry (OCP) (3, 5). However, it has been shown that intraoperative measurement (immediately after the ablation procedure) may lead to underestimation of the residual stromal bed thickness because of the stromal dehydration (3, 5, 25, 26). To reduce the effect of the stromal dehydration, we chose to measure the corneal thickness before and after surgery. Several investigations achieved excellent repeatability and reproducibility of Pentacam, Sirius, and RTVue OCT measurements of the corneal thickness in both healthy and post-refractive-surgery subjects (9, 12, 13, 27–30). We, therefore, performed all measurements with these three instruments to avoid any bias related to the use of one device only.

Our results at one month show that in the corneal center and in the locations at 1 mm from the center, all postop-AD values were larger than the predicted AD ones, whereas at 2.5 mm from the center, almost all postop-AD values were lower than the predicted AD. The laser energy may contribute to the discrepancy. The central cornea was flatter and the cosine effect was reduced, the laser ablation efficacy may be increased. Besides, the ablation depth is larger in the central region than that in the peripheral. It means the treatment requires more times in the central area, the cornea becomes more dehydrated and the laser ablation rate increases over the time. Similar results were found in a previous study, the predicted AD was higher than the measured AD at corneal vertex in the less than 63.49 μm group and lower in the greater than 63.49 μm group (11). At three months the postop-AD decreased, indicating an increase in the corneal thickness during this period, that decreased the Δ-AD values from negative to close to zero. Consistently, at 2.5 mm from the center, all three instruments showed a change of the Δ-AD values from slightly positive to markedly positive, which again revealed a decrease in the postop-AD. We suppose that epithelial remodeling and thickening contributes to the narrowing of the gap between the predicted AD and postop-AD, especially in the central and paracentral cornea. Several studies support this explanation of the observed phenomenon (10, 31–33). Nonetheless, no clear explanation has been proposed for midperipheral thickening, which requires further research.

The 95% LoA are important also in the estimation of the reliability of the predicted AD. In our examination, their values were within a relatively wide range, as the maximum absolute values in the central 1-mm region were approximately 20 μm and roughly 30 μm in the 2.5-mm region. The agreement between the predicted AD and the postop-AD obtained by RTVue OCT was the highest as compared to those established by Pentacam and Sirius. However, the 95% LoA range became narrower at three months than those at one month in the current study, our results show that agreement between predicted AD and postop-AD has to be considered moderate.

Using FS200 femtosecond and EX500 excimer lasers (Alcon Laboratories, Inc., Ft. Worth, TX, United States), Savini et al. reported no difference between the average predicted AD and postop-AD values in the central cornea determined by Pentacam (although opposite findings were obtained in eyes with low and high myopic corrections) (11). The myopic corrections were correlate to the AD, but it did not had significant influence on the results. Similar results were published by Kanellopoulos et al. who used the same lasers, as well as by Febbraro et al. (7, 10). These findings are in accordance with our postoperative data obtained at three months. The ΔAD is different in different position (superior or interior, nasal or temporal). The mean cylindrical correction of the patients enrolled was −0.79 ± 0.60 D and predicted AD showed no obvious difference in different position. We thought the major cause may be the corneal and epithelial remodeling and thickening. But further studies would be needed to clarify the effect of high astigmatism and corneal thickness.

Since the results of our comparison between the predicted and the postoperative AD are quite similar with all three devices and suggest an early underestimation of the predicted AD, we reinforce the advice that the highest possible caution should be exercised in the planning of the correction of highly myopic eyes with thin corneas. Nevertheless, our study had some limitations. First, the Pentacam and Sirius could not provide the epithelial thickness, we did not separately analyze the parameters. In the studies of Kanellopoulos AJ, the center epithelial thickness and mean thickness seemed to increase after LASIK by 1.58 ± 2.73 μm and 2.88 ± 3.15 μm at 1-month, respectively. At 12 months, the difference was 1.42 ± 2.62 μm and 2.90 ± 2.73 μm, respectively (34). The epithelial thickness showed a significant increase in the first month post-LASIK, and became relatively stable after that up to 2 years (35). The change of epithelial thickness might not show greatly influence but we should still take caution. Second, our follow-up was limited to only three months. Third, for the restriction of measure principle, the CCT measurements were performed at the corneal vertex while the excimer laser was performed at the optical axis. Thinnest point, corneal vertex and pupil center was all enrolled in the study of Savini, and the results of measured ablation depth were close (11). In myopic eyes with small angle kappa, corneal light reflex nearly coincides with the corneal vertex rather than the pupil center or thinnest point. The values in the corneal center and in the locations at 1 mm from the center were all analyzed and similar results were gained. We thought eccentricity may affect the real central values but not have significant influence on the conclusion.

In conclusion, we observed that the average predicted AD in the eyes with myopic FS-LASIK was underestimated in the central and paracentral cornea in early stage. That underestimation progressively decreased from one to three months, probably because of corneal remodeling. Both Scheimpflug imaging and OCT imaging are valuable and reliable for corneal thickness assessment as they provided similar information.

## Data Availability Statement

The raw data supporting the conclusions of this article will be made available by the authors, without undue reservation.

## Ethics Statement

The studies involving human participants were reviewed and approved by the Ethics Committee of the Eye Hospital of Wenzhou Medical University (Wenzhou, Zhejiang, China). The patients/participants provided their written informed consent to participate in this study.

## Author Contributions

HC, QW, AY, and SC contributed to conception and design of the study. ZW, KL, YW, and XL organized the database. HC and KL performed the statistical analysis. ZW wrote the first draft of the manuscript. YW, XL, LD, and ML wrote sections of the manuscript. GS, QW, AY, and SC revised the article critically. All authors contributed to manuscript revision, read, and approved the submitted version.

## Conflict of Interest

The authors declare that the research was conducted in the absence of any commercial or financial relationships that could be construed as a potential conflict of interest.

## Publisher’s Note

All claims expressed in this article are solely those of the authors and do not necessarily represent those of their affiliated organizations, or those of the publisher, the editors and the reviewers. Any product that may be evaluated in this article, or claim that may be made by its manufacturer, is not guaranteed or endorsed by the publisher.
