# Supplementary material for: Agreement Between Predicted and Actual Measured Ablation Depth After FS-LASIK Using Different Rotating Scheimpflug Cameras and OCT
Source: Front Med (Lausanne). 2022 May 19;9:907334. doi: 10.3389/fmed.2022.907334 (PMC9160334; doi:10.3389/fmed.2022.907334)
Supplement: Supplementary file 1 [file Table_1.DOCX]

| Table S1. Mean difference, results of the paired T-test, and 95% limits of agreement (LoA) for differences (ΔAD) between the predicted ablation depth and the postoperative ablation depth determined by the Pentacam HR at one month postoperatively (N = 42) | | | |
| --- | --- | --- | --- |
| Parameters | Mean Difference ± SD | *P* Value | 95% LoA |
| ΔAD_C_ | -8.05±7.81 | <0.001 | -23.4 to 7.3 |
| ΔAD_S-1mm_ | -8.13±11.30 | <0.001 | -30.3 to 14.0 |
| ΔAD_I-1mm_ | -1.49±7.87 | 0.252 | -16.9 to 13.9 |
| ΔAD_N-1mm_ | -6.80±8.82 | <0.001 | -24.1 to 10.5 |
| ΔAD_T-1mm_ | -5.68±9.07 | <0.001 | -23.5 to 12.1 |
| ΔAD_S-2.5mm_ | -3.67±18.21 | 0.222 | -39.4 to 32.0 |
| ΔAD_I-2.5mm_ | 8.68±10.61 | <0.001 | -12.1 to 29.5 |
| ΔAD_N-2.5mm_ | 0.99±10.95 | 0.582 | -20.5 to 22.5 |
| ΔAD_T-2.5mm_ | 2.97±10.57 | 0.091 | -17.7 to 23.7 |
| ΔAD = predicted AD minus postop-AD. | | | |
