# Supplementary material for: Agreement Between Predicted and Actual Measured Ablation Depth After FS-LASIK Using Different Rotating Scheimpflug Cameras and OCT
Source: Front Med (Lausanne). 2022 May 19;9:907334. doi: 10.3389/fmed.2022.907334 (PMC9160334; doi:10.3389/fmed.2022.907334)
Supplement: Supplementary file 2 [file Table_2.DOCX]

| Table S2. Mean difference, results of the paired T-test, and 95% limits of agreement (LoA) for differences (ΔAD) between the predicted ablation depth and the postoperative ablation depth determined by the Sirius at one month postoperatively (N = 42) | | | |
| --- | --- | --- | --- |
| Parameters | Mean Difference ± SD | *P* Value | 95% LoA |
| ΔAD_C_ | -13.21±9.12 | <0.001 | -31.1 to 4.7 |
| ΔAD_S-1mm_ | -8.71±9.52 | <0.001 | -27.4 to 10.0 |
| ΔAD_I-1mm_ | -5.61±7.99 | <0.001 | -21.3 to 10.1 |
| ΔAD_N-1mm_ | -8.82±8.31 | <0.001 | -25.1 to 7.5 |
| ΔAD_T-1mm_ | -8.76±8.31 | <0.001 | -25.0 to 7.5 |
| ΔAD_S-2.5mm_ | 4.49±11.38 | 0.020 | -17.8 to 26.8 |
| ΔAD_I-2.5mm_ | 12.34±9.53 | <0.001 | -6.3 to 31.0 |
| ΔAD_N-2.5mm_ | 5.58±9.50 | <0.001 | -13.0 to 24.2 |
| ΔAD_T-2.5mm_ | 5.37±8.36 | ＜0.001 | -11.0 to 21.8 |
| ΔAD = predicted AD minus postop-AD. | | | |
