# Supplementary material for: Agreement Between Predicted and Actual Measured Ablation Depth After FS-LASIK Using Different Rotating Scheimpflug Cameras and OCT
Source: Front Med (Lausanne). 2022 May 19;9:907334. doi: 10.3389/fmed.2022.907334 (PMC9160334; doi:10.3389/fmed.2022.907334)
Supplement: Supplementary file 3 [file Table_3.DOCX]

| Table S3. Mean difference, results of the paired T-test, and 95% limits of agreement (LoA) for differences (ΔAD) between the predicted ablation depth and the postoperative ablation depth determined by the RTVue OCT at one month postoperatively (N = 42) | | | |
| --- | --- | --- | --- |
| Parameters | Mean Difference ± SD | *P* Value | 95% LoA |
| ΔAD_C_ | -6.89±7.60 | <0.001 | -21.8 to 8.0 |
| ΔAD_S-1mm_ | -7.03±9.23 | <0.001 | -25.1 to 11.1 |
| ΔAD_I-1mm_ | -1.01±6.26 | 0.325 | -13.3 to 11.3 |
| ΔAD_N-1mm_ | -4.20±7.09 | 0.001 | -18.1 to 9.7 |
| ΔAD_T-1mm_ | -4.32±7.01 | 0.001 | -18.1 to 9.4 |
| ΔAD_S-2.5mm_ | 0.82±8.99 | 0.579 | -16.8 to 18.4 |
| ΔAD_I-2.5mm_ | 8.03±7.95 | <0.001 | -7.5 to 23.6 |
| ΔAD_N-2.5mm_ | 4.96±8.01 | <0.001 | -10.7 to 20.7 |
| ΔAD_T-2.5mm_ | 3.58±6.50 | 0.002 | -9.2 to 16.3 |
| ΔAD = predicted AD minus postop-AD. | | | |
