# Supplementary material for: Agreement Between Predicted and Actual Measured Ablation Depth After FS-LASIK Using Different Rotating Scheimpflug Cameras and OCT
Source: Front Med (Lausanne). 2022 May 19;9:907334. doi: 10.3389/fmed.2022.907334 (PMC9160334; doi:10.3389/fmed.2022.907334)
Supplement: Supplementary file 4 [file Table_4.DOCX]

| Table S4. Mean difference, results of the paired T-test, and 95% limits of agreement (LoA) for differences (ΔAD) between the predicted ablation depth and the postoperative ablation depth determined by the Pentacam HR at 3 months postoperatively (N = 42) | | | |
| --- | --- | --- | --- |
| Parameters | Mean Difference ± SD | *P* Value | 95% LoA |
| ΔAD_C_ | 0.67±9.39 | 0.648 | -17.7 to 19.1 |
| ΔAD_S-1mm_ | -0.35±12.24 | 0.856 | -24.3 to 23.6 |
| ΔAD_I-1mm_ | 7.14±8.67 | <0.001 | -9.8 to 24.1 |
| ΔAD_N-1mm_ | 0.45±10.10 | 0.773 | -19.3 to 20.2 |
| ΔAD_T-1mm_ | 3.77±10.39 | 0.023 | -16.6 to 24.1 |
| ΔAD_S-2.5mm_ | 4.15±19.25 | 0.169 | -33.6 to 41.9 |
| ΔAD_I-2.5mm_ | 15.26±10.45 | <0.001 | -5.2 to 35.8 |
| ΔAD_N-2.5mm_ | 6.29±13.98 | 0.006 | -21.1 to 33.7 |
| ΔAD_T-2.5mm_ | 10.89±13.21 | <0.001 | -15.0 to 36.8 |
| ΔAD = predicted AD minus postop-AD. | | | |
