# Supplementary material for: Agreement Between Predicted and Actual Measured Ablation Depth After FS-LASIK Using Different Rotating Scheimpflug Cameras and OCT
Source: Front Med (Lausanne). 2022 May 19;9:907334. doi: 10.3389/fmed.2022.907334 (PMC9160334; doi:10.3389/fmed.2022.907334)
Supplement: Supplementary file 5 [file Table_5.DOCX]

| Table S5. Mean difference, results of the paired T-test, and 95% limits of agreement (LoA) for differences (ΔAD) between the predicted ablation depth and the postoperative ablation depth determined by the Sirius at 3 months postoperatively (N = 42) | | | |
| --- | --- | --- | --- |
| Parameters | Mean Difference ± SD | *P* Value | 95% LoA |
| ΔAD_C_ | -7.92±9.05 | <0.001 | -25.7 to 9.8 |
| ΔAD_S-1mm_ | -5.12±10.24 | 0.002 | -25.2 to 14.9 |
| ΔAD_I-1mm_ | -0.25±9.45 | 0.865 | -18.8 to 18.3 |
| ΔAD_N-1mm_ | -4.73±8.58 | 0.001 | -21.5 to 12.1 |
| ΔAD_T-1mm_ | -2.99±9.22 | 0.042 | -21.1 to 15.1 |
| ΔAD_S-2.5mm_ | 5.90±14.08 | 0.010 | -21.7 to 33.5 |
| ΔAD_I-2.5mm_ | 15.73±9.75 | <0.001 | -3.4 to 34.8 |
| ΔAD_N-2.5mm_ | 7.33±10.02 | <0.001 | -12.3 to 27.0 |
| ΔAD_T-2.5mm_ | 10.05±10.26 | <0.001 | -10.1 to 30.1 |
| ΔAD = predicted AD minus postop-AD. | | | |
