# Supplementary material for: Agreement Between Predicted and Actual Measured Ablation Depth After FS-LASIK Using Different Rotating Scheimpflug Cameras and OCT
Source: Front Med (Lausanne). 2022 May 19;9:907334. doi: 10.3389/fmed.2022.907334 (PMC9160334; doi:10.3389/fmed.2022.907334)
Supplement: Supplementary file 6 [file Table_6.DOCX]

| Table S6. Mean difference, results of the paired T-test, and 95% limits of agreement (LoA) for differences (ΔAD) between the predicted ablation depth and the postoperative ablation depth determined by the RTVue OCT at 3 months postoperatively (N = 42) | | | |
| --- | --- | --- | --- |
| Parameters | Mean Difference ± SD | *P* Value | 95% LoA |
| ΔAD_C_ | -1.36±8.31 | 0.296 | -17.7 to 14.9 |
| ΔAD_S-1mm_ | -1.40±8.84 | 0.309 | -18.7 to 15.9 |
| ΔAD_I-1mm_ | 5.20±7.24 | <0.001 | -9.0 to 19.4 |
| ΔAD_N-1mm_ | 0.37±7.45 | 0.750 | -14.2 to 15.0 |
| ΔAD_T-1mm_ | 1.55±8.43 | 0.241 | -15.0 to 18.1 |
| ΔAD_S-2.5mm_ | 6.13±11.01 | 0.001 | -15.4 to 27.7 |
| ΔAD_I-2.5mm_ | 12.94±8.34 | <0.001 | -3.4 to 29.3 |
| ΔAD_N-2.5mm_ | 7.31±9.52 | <0.001 | -11.3 to 26.0 |
| ΔAD_T-2.5mm_ | 8.81±7.50 | <0.001 | -5.9 to 23.5 |
| ΔAD = predicted AD minus postop-AD. | | | |
