# Supplementary material for: Agreement Between Predicted and Actual Measured Ablation Depth After FS-LASIK Using Different Rotating Scheimpflug Cameras and OCT
Source: Front Med (Lausanne). 2022 May 19;9:907334. doi: 10.3389/fmed.2022.907334 (PMC9160334; doi:10.3389/fmed.2022.907334)
Supplement: Supplementary file 7 [file Presentation_1.pdf]

Figure S1. Bland-Altman plots of the predicted ablation depth against actual measured corneal ablation depth using the Pentacam HR in postoperative 1 month. The solid line represents the mean difference. The dotted lines on the side represent the upper and lower 95% confidence interval limits of agreement. (A) agreement of central corneal thickness; (B) agreement at a distance of superior 1.0 mm from the corneal vertex; (C) agreement at inferior 1.0 mm from the corneal vertex; (D) agreement at nasal 1.0 mm from the corneal vertex; (E) agreement at temporal 1.0 mm from the corneal vertex; (F) agreement at a distance of superior 2.5 mm from the corneal vertex; (G) agreement at a distance of inferior 2.5 mm from the corneal vertex; (H) agreement at a distance of nasal 2.5 mm from the corneal vertex; (I) agreement at a distance of temporal 2.5 mm from the corneal vertex.

Figure S2. Bland-Altman plots of the predicted ablation depth against actual measured corneal ablation depth using the Pentacam HR in postoperative 3 months. The solid line represents the mean difference. The dotted lines on the side represent the upper and lower 95% confidence interval limits of agreement. (A) agreement of central corneal thickness; (B) agreement at a distance of superior 1.0 mm from the corneal vertex; (C) agreement at inferior 1.0 mm from the corneal vertex; (D) agreement at nasal 1.0 mm from the corneal vertex; (E) agreement at temporal 1.0 mm from the corneal vertex; (F) agreement at a distance of superior 2.5 mm from the corneal vertex; (G) agreement at a distance of inferior 2.5 mm from the corneal vertex; (H) agreement at a distance of nasal 2.5 mm from the corneal vertex; (I) agreement at a distance of temporal 2.5 mm from the corneal vertex.

Figure S3. Bland-Altman plots of the predicted ablation depth against actual measured

corneal ablation depth using the Sirius in postoperative 1 month. The solid line represents the mean difference. The dotted lines on the side represent the upper and lower 95% confidence interval limits of agreement. (A) agreement of central corneal thickness; (B) agreement at a distance of superior 1.0 mm from the corneal vertex; (C) agreement at inferior 1.0 mm from the corneal vertex; (D) agreement at nasal 1.0 mm from the corneal vertex; (E) agreement at temporal 1.0 mm from the corneal vertex; (F) agreement at a distance of superior 2.5 mm from the corneal vertex; (G) agreement at a distance of inferior 2.5 mm from the corneal vertex; (H) agreement at a distance of nasal 2.5 mm from the corneal vertex; (I) agreement at a distance of temporal 2.5 mm from the corneal vertex.

Figure S4. Bland-Altman plots of the predicted ablation depth against actual measured corneal ablation depth using the Sirius in postoperative 3 months. The solid line represents the mean difference. The dotted lines on the side represent the upper and lower 95% confidence interval limits of agreement. (A) agreement of central corneal thickness; (B) agreement at a distance of superior 1.0 mm from the corneal vertex; (C) agreement at inferior 1.0 mm from the corneal vertex; (D) agreement at nasal 1.0 mm from the corneal vertex; (E) agreement at temporal 1.0 mm from the corneal vertex; (F) agreement at a distance of superior 2.5 mm from the corneal vertex; (G) agreement at a distance of inferior 2.5 mm from the corneal vertex; (H) agreement at a distance of nasal 2.5 mm from the corneal vertex; (I) agreement at a distance of temporal 2.5 mm from the corneal vertex.

Figure S5. Bland-Altman plots of the predicted ablation depth against actual measured corneal ablation depth using the RTVue OCT in postoperative 1 month. The solid line

represents the mean difference. The dotted lines on the side represent the upper and lower 95% confidence interval limits of agreement. (A) agreement of central corneal thickness; (B) agreement at a distance of superior 1.0 mm from the corneal vertex; (C) agreement at inferior 1.0 mm from the corneal vertex; (D) agreement at nasal 1.0 mm from the corneal vertex; (E) agreement at temporal 1.0 mm from the corneal vertex; (F) agreement at a distance of superior 2.5 mm from the corneal vertex; (G) agreement at a distance of inferior 2.5 mm from the corneal vertex; (H) agreement at a distance of nasal 2.5 mm from the corneal vertex; (I) agreement at a distance of temporal 2.5 mm from the corneal vertex.

Figure S6. Bland-Altman plots of the predicted ablation depth against actual measured corneal ablation depth using the RTVue OCT in postoperative 3 months. The solid line represents the mean difference. The dotted lines on the side represent the upper and lower 95% confidence interval limits of agreement. (A) agreement of central corneal thickness; (B) agreement at a distance of superior 1.0 mm from the corneal vertex; (C) agreement at inferior 1.0 mm from the corneal vertex; (D) agreement at nasal 1.0 mm from the corneal vertex; (E) agreement at temporal 1.0 mm from the corneal vertex; (F) agreement at a distance of superior 2.5 mm from the corneal vertex; (G) agreement at a distance of inferior 2.5 mm from the corneal vertex; (H) agreement at a distance of nasal 2.5 mm from the corneal vertex; (I) agreement at a distance of temporal 2.5 mm from the corneal vertex.
