# Supplementary figures and images for: Agreement Between Predicted and Actual Measured Ablation Depth After FS-LASIK Using Different Rotating Scheimpflug Cameras and OCT
Source: Front Med (Lausanne). 2022 May 19;9:907334. doi: 10.3389/fmed.2022.907334 (PMC9160334; doi:10.3389/fmed.2022.907334)

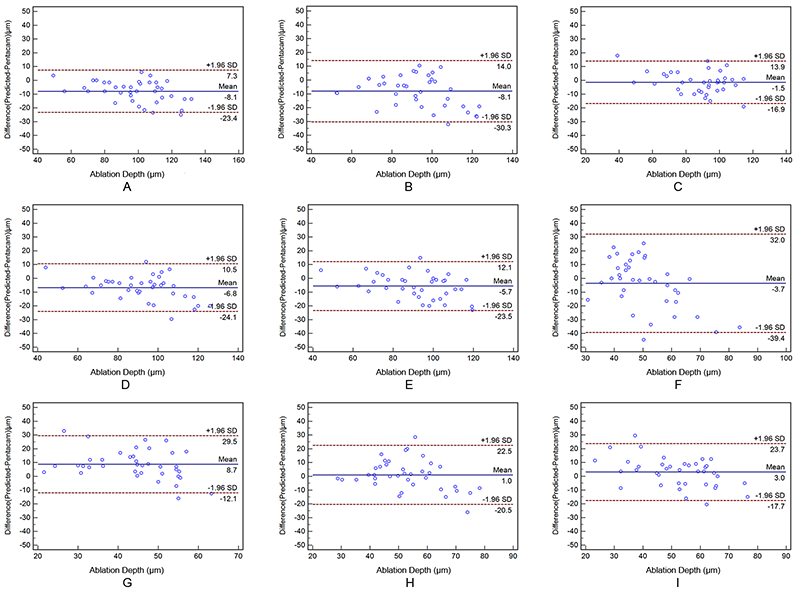

Supplement: Supplementary file 8 [file Image_1.TIF]

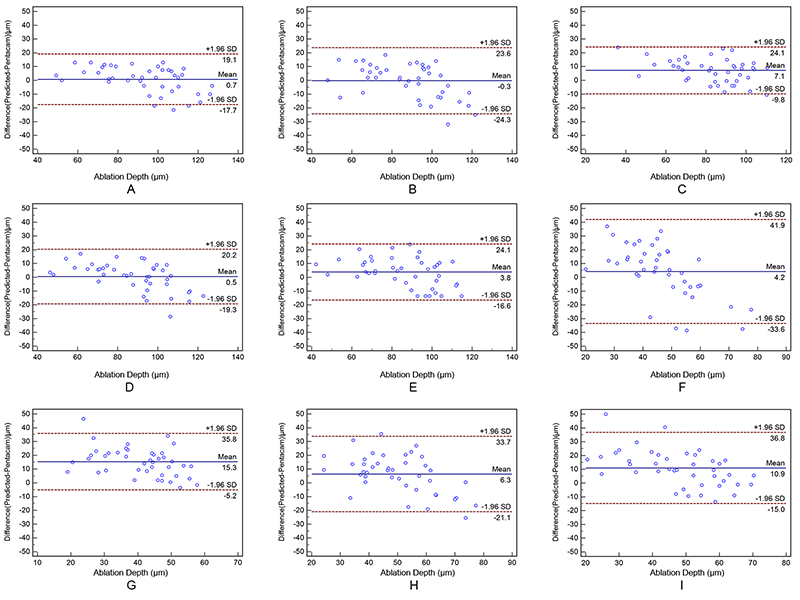

Supplement: Supplementary file 9 [file Image_2.TIF]

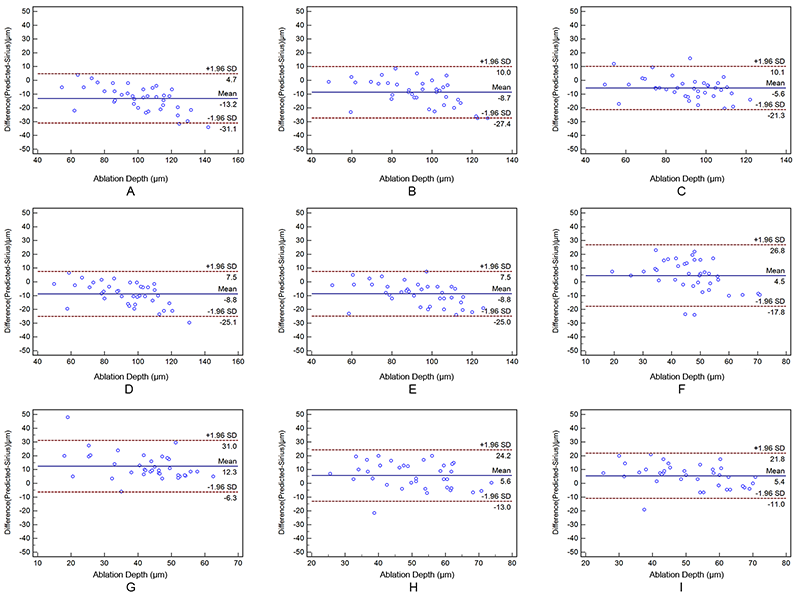

Supplement: Supplementary file 10 [file Image_3.TIF]

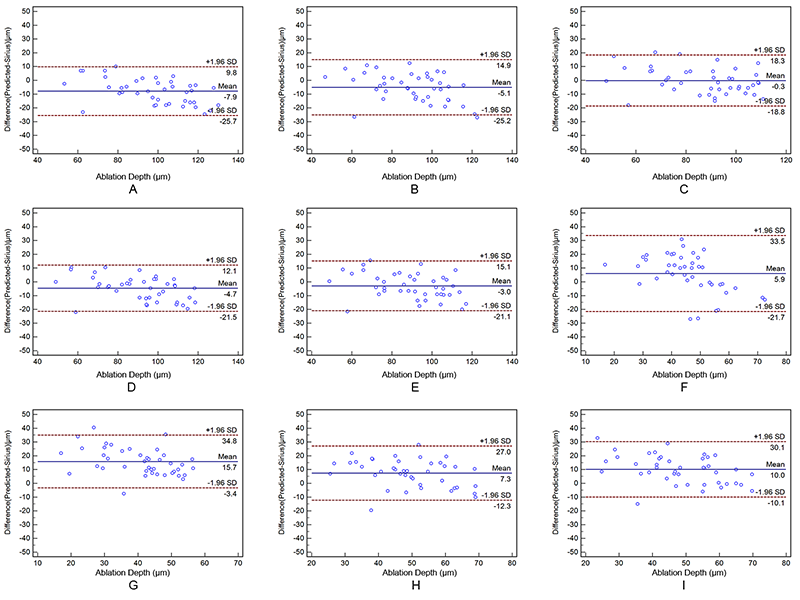

Supplement: Supplementary file 11 [file Image_4.TIF]

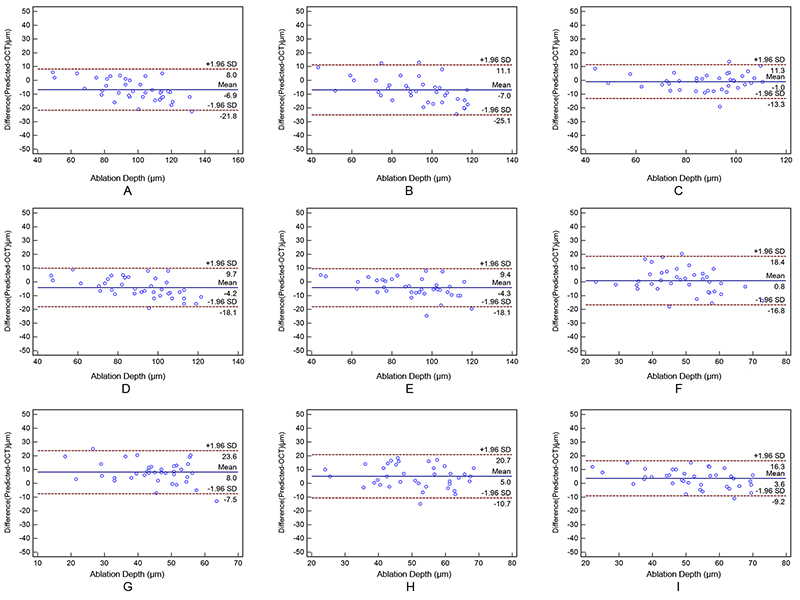

Supplement: Supplementary file 12 [file Image_5.TIF]

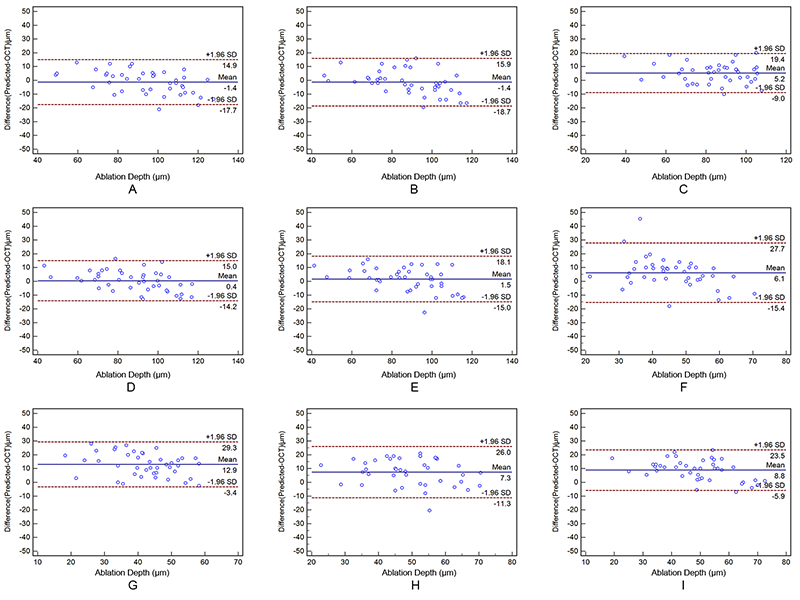

Supplement: Supplementary file 13 [file Image_6.TIF]
